# The Neural Basis of Approach-Avoidance Conflict: A Model Based Analysis

**DOI:** 10.1523/ENEURO.0115-19.2019

**Published:** 2019-08-07

**Authors:** Samuel Zorowitz, Alexander P. Rockhill, Kristen K. Ellard, Katherine E. Link, Todd Herrington, Diego A. Pizzagalli, Alik S. Widge, Thilo Deckersbach, Darin D. Dougherty

**Affiliations:** 1 Division of Neurotherapeutics, Department of Psychiatry, Massachusetts General Hospital, Charlestown, MA 02129; 2 Department of Neurology, Massachusetts General Hospital, Boston, MA 02114; 3 Department of Psychiatry, McLean Hospital, Belmont, MA 02478

**Keywords:** approach-avoidance, cognitive, decision making, fMRI, psychiatry, psychology

## Abstract

Approach-avoidance conflict arises when the drives to pursue reward and avoid harm are incompatible. Previous neuroimaging studies of approach-avoidance conflict have shown large variability in reported neuroanatomical correlates. These prior studies have generally neglected to account for potential sources of variability, such as individual differences in choice preferences and modeling of hemodynamic response during conflict. In the present study, we controlled for these limitations using a hierarchical Bayesian model (HBM). This enabled us to measure participant-specific per-trial estimates of conflict during an approach-avoidance task. We also employed a variable epoch method to identify brain structures specifically sensitive to conflict. In a sample of 28 human participants, we found that only a limited set of brain structures [inferior frontal gyrus (IFG), right dorsolateral prefrontal cortex (dlPFC), and right pre-supplementary motor area (pre-SMA)] are specifically correlated with approach-avoidance conflict. These findings suggest that controlling for previous sources of variability increases the specificity of the neuroanatomical correlates of approach-avoidance conflict.

## Significance Statement

Approach-avoidance conflict is implicated in many psychiatric syndromes. Previous fMRI studies of this important process have potential biases caused by overlooking individual differences in the evaluation of reward and threat in their analyses. We present a method to model individual differences in approach-avoidance conflict and demonstrate how to incorporate this model into fMRI analyses. We found our approach to have greater specificity than previous studies, which highlights the importance of capturing large variability in participant behavior.

## Introduction

The drive for self-preservation is fundamental to every living organism. Behavioral psychologists have long argued that animals evaluate objects and events in their environments along an appetitive-aversive continuum (Elliot, 2008; Corr, 2013), where animals are motivated to approach things that sustain them (e.g., rewarding or pleasurable stimuli) and to avoid things that threaten them (e.g., harmful or painful stimuli). Approach-avoidance conflict arises in situations where these drives are incompatible, such as when the approach toward reward also increases the possibility of danger. Approach-avoidance conflict is an important phenomenon as it is thought to be core to the etiology and maintenance of psychiatric disorders including depression and anxiety (American Psychiatric Association, 2013).

In recent years, many studies have investigated the neural substrates underlying approach-avoidance conflict using electrophysiology in rodents (Friedman et al., 2015) and non-human primates (Amemori et al., 2015) and neuroimaging in humans (Talmi et al., 2009; Park et al., 2011; Bach et al., 2014; Aupperle et al., 2015; O’Neil et al., 2015; Schlund et al., 2016; Loh et al., 2017). The results of the human neuroimaging literature have implicated a diverse collection of brain structures in approach-avoidance conflict including cortical structures such as the anterior cingulate, insula, orbitofrontal cortex, and dorsolateral prefrontal cortex (dlPFC), and subcortical structures including the amygdala, hippocampus, and striatum. There is considerable heterogeneity in these findings, however, such that none of the aforementioned brain structures are consistently identified as being involved in approach-avoidance conflict across these studies. This naturally prompts the question of where some of the variability might stem from.

One possibility is that the heterogeneity reflects variability in approach-avoidance behavior across participants. Approach-avoidance tendencies are naturally varying across individuals (Carver and White, 1994), such that there are robust individual differences in the valuation of reward and threat cues. As such, the point of maximal approach-avoidance conflict is unlikely to be the same across participants. Ignoring these individual differences and averaging across them, however, has been shown to reduce contrast statistics in fMRI group level analysis (Ahn et al., 2011). One solution is to explicitly model individual differences in approach-avoidance conflict, such as with hierarchical Bayesian modeling (HBM; Kruschke, 2015), and incorporate trial-by-trial estimates of approach-avoidance conflict into the fMRI analysis to align participants along a latent evaluation space (O’Doherty et al., 2007; Ahn et al., 2011). In doing so, we are less likely to average out conflict-related changes in BOLD signal.

A second possibility is that the heterogeneity in findings directly reflects variability in previous modeling of conflict-related changes in BOLD signal. A hallmark feature of approach-avoidance conflict is prolonged reaction times. Interpreting changes in BOLD signal between two conditions that also involve differences in response times is challenging, however, due to the time-on-task effect (Taylor et al., 2014). Because the BOLD signal sums approximately linearly as a function of stimulation duration (Dale and Buckner, 1997), brain structures not directly involved in the representation of approach-avoidance conflict may still show increases in BOLD signal by virtue of prolonged processing of the constitutive elements of conflict (e.g., rewarding or threatening stimuli). Controlling for response time is necessary then to identify brain structures that are directly involved in the processing of approach-avoidance conflict (brain regions that show increased intensity of activity, not just prolonged activity). With the exception of Talmi et al. (2009), the neuroimaging studies of approach-avoidance conflict cited above do not document having incorporated response times into their fMRI analyses.

In the present study, we investigated the neural signatures of human approach-avoidance conflict with functional neuroimaging controlling for the issues discussed above. We measured changes in the fMRI BOLD signal as participants completed an approach-avoidance conflict task. In the task, participants repeatedly chose between a risky option, returning greater reward at the risk of potential electrical stimulation, and a safe option, returning a much smaller reward but no risk of electrical stimulation. Using a novel HBM, we estimated participants’ per-trial approach-avoidance conflict and used these to inform our fMRI analyses. Moreover, we controlled for the time-on-task effect using the variable epoch method (Grinband et al., 2008) to identify brain structures that showed greater intensity of activity, rather than prolonged activity, during approach-avoidance conflict. We found that using these methods increased the specificity of the structures responding to conflict.

## Materials and Methods

### Subjects

Thirty-six individuals (13 females, 23 males, age: mean = 33.94 years, SD = 8.80) were recruited from the Greater Boston area to participate as healthy volunteers in a research program to develop novel deep brain stimulation (DBS) technologies (Widge et al., 2017). All participants reported being right-handed and without a current or past diagnosis of a psychiatric or neurologic disorder and were in the normal healthy range for the Mini-International Neuropsychiatric Interview (MINI; Sheehan et al., 1998). Women were scanned at or near the ovulation phase of their menstrual cycles (when estradiol is lowest) to minimize potential gender confounds (Zeidan et al., 2011). The study was approved by the Partners Health care System Human Research Committee, and all participants provided written informed consent before enrollment. Participants were paid $600 for the successful completion of the larger study protocol.

Eight individuals were excluded from analysis: five due to technical complications (see Task below), two for missing responses for >20% of trials, and one due to corrupted DICOMs. This resulted in a final sample of 28 participants (10 females, 18 males).

### Task

**Figure 1.**
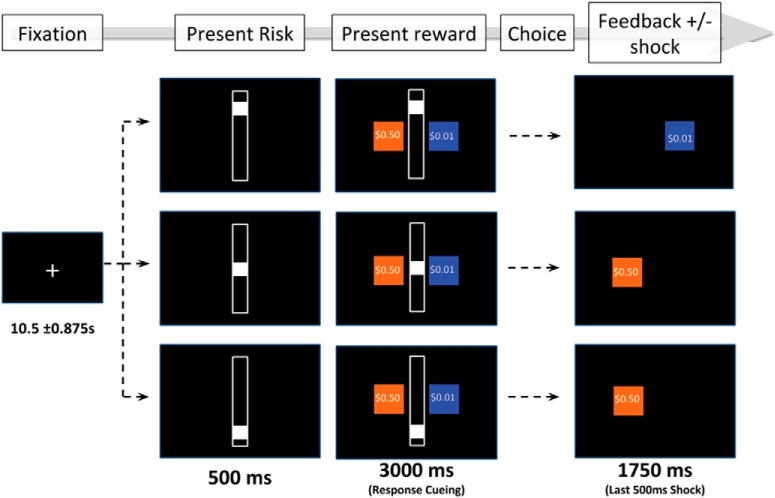
Aversion-reward conflict (ARC) task. Participants are presented with a safe choice (blue) and a risky choice (orange). The safe choice pays a guaranteed small reward ($0.01) and no aversive stimulation. The risky choice pays a guaranteed larger reward ($0.05–$0.95), and a probability of stimulation as indicated by the centered white bar. Participants decide whether to accept a higher payout at risk of aversive stimulation. *Figure Contributions*: Darin Dougherty, Thilo Deckersbach, Alik Widge, and Samuel Zorowitz designed the task. Sam Zorowitz created the figure.

We employed a modified version of the aversion-reward conflict (ARC) task (Sierra-Mercado et al., 2015). During this task, participants make a series of choices between two options: a safe option or a risky option (Fig. 1). Selecting the safe option returns a reward of $0.01, and the participant never receives electrical stimulation. In contrast, selecting the risky option returns a reward between $0.05 and $0.95, and the participant receives electrical stimulation with probability 10%, 50%, or 90%, as indicated by a bar in the center of the screen. This required participants to evaluate their preference for a greater reward with a risk of electrical stimulation relative to a lesser reward with no risk of electrical stimulation. Participants were instructed to choose as fast as possible without choosing randomly and were informed that their choices would be reflected in their final study payment. (In fact, each participant was compensated with a generous flat payment.) Before starting the task, participants were asked to report back the instructions so that their comprehension could be verified. Next, participants completed ten practice trials to become accustomed to the timing of the task.

This ARC task had three levels of risk: 10%, 50%, and 90% likelihood of electrical stimulation. Rewards were sampled from all cent values between $0.05 and $0.95. Trials were counterbalanced such that there were an equal number of trials at each risk level, while rewards were uniformly and equally sampled within each risk level. Each participant completed 108 total trials and the order of trials was kept constant for all the participants. Long intertrial intervals of 10.5 ± 0.875 s separated sequential trials in the task (a slow event-related design). The duration of the full task was 28.5 min.

Electrical stimulation was administered to the ankle through a Coulbourn Aversive Finger Stimulator (Harvard Apparatus, E13-22; maximum level of stimulation = 4.0 mA). The amperage of electrical stimulation was calibrated individually for each participant before performing the ARC task. Participants experienced increasing levels of stimulation until they reported reaching a subjective threshold qualified as “highly annoying but not painful.” For five participants this threshold could not be established because the highest stimulation setting of 4.0 mA was too painful, but penultimate 2.3-mA setting was not experienced as annoying. These participants did not exhibit behavioral variation (i.e., they always accepted the risky choice) and consequently these participants were excluded from the analysis.

### Behavioral analysis

Our aim was to infer the level of approach-avoidance conflict experienced by each participant during every trial. We devised a novel HBM that predicts participants’ choices (safe or risky option) and response times. The decision to model response times was motivated by well-documented relationship between decision conflict and prolonged response times and prior demonstrations that including response times in behavioral models improved the accuracy of single-trial parameter estimation (Prerau et al., 2009; Pedersen et al., 2017). The model is composed of a logistic regression on the choice data and a gamma regression on the response times. We assume the binary choice responses, y ∈ (0 = safe choice, 1 = risky choice) are drawn from the Bernoulli distribution:

$$p\left(y_{ij}\text{|}\theta_{ij}\right)=\theta_{ij}^{y_{ij}}{\left(1-\theta_{ij}\right)}^{1-y_{ij}},$$

where $\theta_{ij}$ is the likelihood-of-take for trial *i* and participant *j*, and is itself estimated from:

$$\theta_{ij}=logistic\left(\beta_{0_{j}}+\Sigma \beta_{nj}x_{nij}\right).$$

Here, $\beta_{0_{j}}$is the intercept for participant *j*; the remaining $\beta_{nj}$ regression coefficients reflect the modulatory influence of independent variables, *X,* on the baseline likelihood-of-take. In this model, there are three independent variables: 50% risk$\left(\beta_{1}\right)$, 90% risk$\left(\beta_{2}\right)$, and reward$\left(\beta_{3}\right)$. The 50% risk $\left(\beta_{1}\right)$ and 90% risk $\left(\beta_{2}\right)$, coefficients are binary predictors, whereas reward $\left(\beta_{3}\right)$ is a continuous predictor that was normalized to have mean = 0 and SD = 1. The intercept term, $\beta_{0}$, thus reflects the likelihood of take for 10% risk and $0.50 reward offer.

The continuous response times, *z*, are assumed to be drawn from the gamma distribution:

$$p\left(Z_{ij}\text{|}k_{j},\mu_{ij}\right)=Gamma\left(k_{j},\frac{k_{j}}{\mu_{ij}}\right),$$

where $k_{j}$ is the shape parameter for participant *j* and $\mu_{ij}$ is the mean of the distribution predicted by:

$$\mu_{ij}=\alpha_{0j}+\alpha_{1j}\cdot d_{ij}.$$

We chose a gamma distribution because it is well-suited for characterizing response times and other strictly positive data with a long rightward tail (Yousefi et al., 2015). Here, $\alpha_{0j}$was the average response time for participant *j* and $\alpha_{1j}$ was the slope term determining how much response time increased with conflict. We represent conflict, $d_{ij}$, as the inverse of the distance-to-decision boundary of trial *i* for participant *j*, represented as:

$$d_{ij}=0.25-{\left(0.5-\theta_{ij}\right)}^{2}.$$

This measure, *d*, has the shape of an inverted parabola. It is greatest when θ=0.5, or when a participant is equally likely to select the safe or risky option. It is smallest when θ=0.0 or θ=1.0, or when a participant is most likely to select the safe or risky option, respectively. Therefore, *d* reflects the degree of conflict a participant experienced during the evaluation phase of a given trial. The model fit then identified the set of parameters that maximized the joint likelihood of both the choice and response time data due to the relationship between θ and *d*.

**Figure 2.**
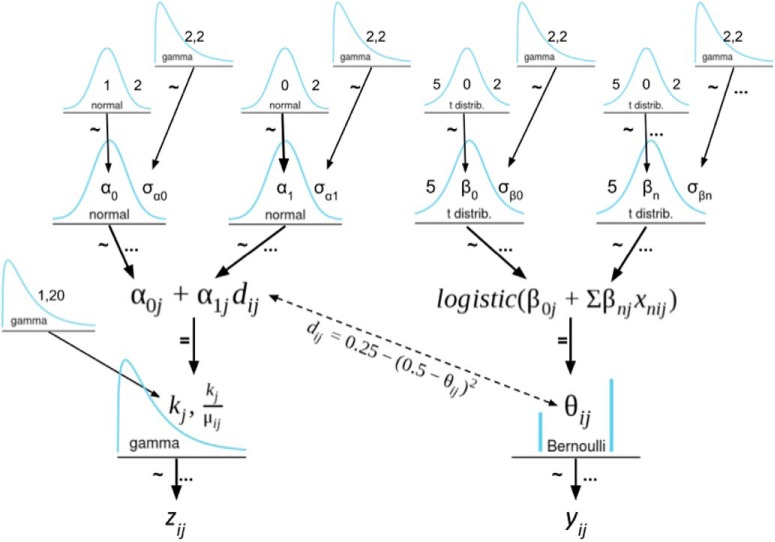
A Kruschke-style diagram of the hierarchical model. The ∼ symbol indicates stochastic dependency, whereas the = symbol indicates a deterministic dependency. Ellipses indicate the indices over which the dependency applies. The parameter of most interest is *d*, the inverse distance-to-decision-boundary, which measures the estimated conflict experienced on a given trial. *Figure Contributions*: Samuel Zorowitz created the model.

As a hierarchical model, each of the participant-level regression parameters defined above (e.g., $\alpha_{0j},\alpha_{1j},\beta_{0j},\beta_{1j},\ldots ,\beta_{nj}$) are drawn a corresponding group-level distribution, centered at group-level means (e.g., $\alpha_{0G},\alpha_{1G},\beta_{0G},\beta_{1G},\ldots ,\beta_{nG}$). Thus, the model simultaneously estimates group- and participant-level parameters, partially pooling the data so as to minimize the influence of outliers. Figure 2 presents a detailed diagram of the model which includes the choice of priors. We assumed Student’s *t* distribution priors on the choice (β) regression coefficients to ensure robust logistic regression (Gelman et al., 2008; Ghosh et al., 2018) using the recommended degrees of freedom, η=5 (Stan Development Team, 2017).

The behavioral model was fit using Hamiltonian Monte Carlo (HMC) sampling in Stan v2.15 (Carpenter et al., 2017) with four chains of 2000 steps each (1000 burn-in, thinning = 4), yielding 1000 posterior samples total. The convergence of the chains was computed using the $\hat{R}$ statistic (Gelman et al., 2014), which measures the degree of variation between chains relative to the variation within chains. The Stan development team recommends as a rule of thumb that all parameters have $\hat{R}$ statistics no >1.1. All parameters in our showed good convergence $\left(\hat{R}\approx 1\right)$. Similarly, the number of effective samples approached 1000 for most parameters indicating that the chains exhibited low autocorrelation. Once fitted, per-trial estimates of *d* were generated by multiplying the observed trial features (risk level and reward value) by the modal individual-level parameter estimates.

### Image acquisition and preprocessing

All MRI scans were completed at the Athinoula A. Martinos Center for Biomedical Imaging. Of the 28 participants included in this analysis, 20 were scanned using a 3T Siemens Trio scanner, and eight were scanned using a 3T Siemens Prisma scanner (scanner type was entered as a covariate in the analyses). All participants were scanned with a 32-channel head coil. Foam cushions were used to restrict head movements. Task images were projected using a rear projection system and PsychToolbox (V3) stimulus presentation software (Kleiner et al., 2007).

For each participant, both structural and functional images were collected. The structural sequences involved a high-resolution, four-multiecho, T1-weighted, magnetization-prepared, gradient-echo image (TR = 2510 ms, TE = 1.64 ms, flip angle = 7°, voxel size = 1.0 × 1.0 × 1.0 mm; van der Kouwe et al., 2008). Functional images were acquired using a multiband SMS-3 T2*-weighted echoplanar imaging (EPI) sequence sensitive to BOLD contrast (TR = 1750 ms, TE = 30 ms, flip angle = 75°, voxel size = 2.0 × 2.0 × 2.0 mm, PAT = GRAPPA, accelerated factor TE = 2). Sixty-three interleaved slices were aligned perpendicular to the plane intersecting the anterior and posterior commissures, and the whole brain was imaged (FOV = 220 mm). For the purpose of EPI-dewarping, a fieldmap was also collected for each participant (63 interleaved slices, TR = 500 ms, TE 1 = 3.41 ms, TE 2 = 5.87 ms, flip angle = 55°, voxel size = 2.0 × 2.0 × 2.0 mm).

Anatomic reconstructions of each participant’s brain were generated from the T1 structural image using Freesurfer v5.3 (Fischl, 2012). The functional data were first corrected for slice timing using the Fourier phase shift interpolation from SPM8 and then for B0 using FSL’s *epidewarp* (https://surfer.nmr.mgh.harvard.edu/fswiki/epidewarp.fsl). FS-FAST v5.3 was used for subsequent preprocessing with their default settings: coregistration with the corresponding Freesurfer anatomic reconstruction; motion correction to the first acquisition using the AFNI motion correction tool (http://afni.nimh.nih.gov/afni/); normalization to fsaverage/Montreal Neurologic Institute (MNI) space; and smoothing using 6-mm FWHM kernel.

### fMRI modeling and analysis

**Figure 3.**
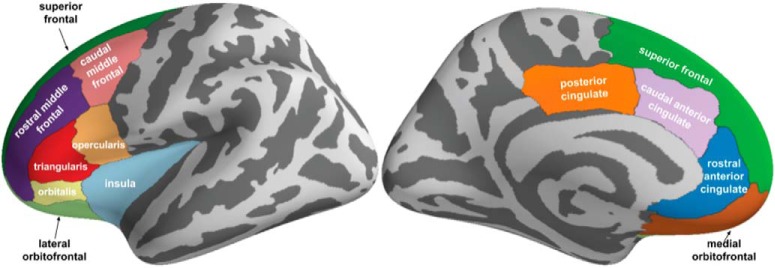
A priori cortical regions of interest. Regions (Freesurfer labels) were selected from the Mindboggle atlas (https://mindboggle.info/data.html) based on the diffuse locations of activations previously reported in the approach-avoidance decision-making literature. *Figure Contributions*: Samuel Zorowitz chose the regions of interest based on prior literature and created the figure.

Neuroimaging analyses were limited to a priori regions of interest in line with the literature (Talmi et al., 2009; Park et al., 2011; Bach et al., 2014; Aupperle et al., 2015; O’Neil et al., 2015; Schlund et al., 2016; Loh et al., 2017). Specifically, a cortical mask was constructed for left and right hemispheres using the Mindboggle atlas (Klein and Tourville, 2012) consisting of areas encompassing the cingulate cortex, dorsomedial PFC (dmPFC), orbitofrontal cortex, dlPFC and ventrolateral PFC, and insular cortex (Fig. 3). Similarly, a subcortical mask was constructed using the automated subcortical segmentation standard in Freesurfer (Fischl et al., 2002) consisting of the bilateral striatum (caudate, putamen), hippocampus, and amygdala.

In the first level analysis, we modeled the deliberation phase (time to response) using the variable epoch method (Grinband et al., 2008). The deliberation phase was modeled using two sets of boxcar regressors: one control regressor and one parametric modulation regressor. For both regressors, the boxcar for each trial was scaled in duration according to that trial’s observed response time. The boxcar for each trial in the parametric modulation term was scaled in amplitude according to estimated decision conflict (*d*) for that trial. The parametric modulation boxcars corresponding to trials with missing responses were scaled to zero amplitude. Additionally, several separate control analyses were performed with the same procedure to determine the effect of (1) using the variable epochs method, (2) using an HBM to model individual differences, and (3) using conflict as the parametric modulator over and above using risk or reward as the parametric modulator. For the first control analysis, fixed epochs were used instead of variable epochs, where the trial duration was not scaled and instead was uniform; from the presentation of the first stimulus (the risk bar) to 3.5 s after that time when subject responses were cutoff. For the second control analysis, an equivalent non-hierarchical model was used (i.e., estimating only group parameters, excluding participant-level parameters) to model the conflict parametric modulator. For the third set of control analyses, risk and reward were used, in separate analyses, to parametrically modulate the deliberation-phase regressor instead of conflict, and, in another separate analysis, risk, reward and conflict were all used as parametric modulators with three parametrically modulated deliberation-phase regressors in the same first-level analysis. All regressors were convolved with the SPM hemodynamic response function. All estimated regression coefficients in first level analysis were converted to percentage signal change (PSC; Pernet, 2014).

The fMRI data were preprocessed using a high-pass filter, nuisance regressors and motion scrubbing. A discrete cosine transform basis set was added to high-pass filter the data at 0.01 Hz. The six possible directions of motion were incorporated into the first-level analyses (after being demeaned, detrended, and orthogonalized) as nuisance regressors. Finally, motion scrubbing was used to mitigate the impact of high-motion acquisitions on the data (Siegel et al., 2014). Volumes for which the calculated framewise displacement (Power et al., 2012) exceeded 0.9 mm were excluded from analyses, and the first four acquisitions were discarded.

In the second level analysis, the beta coefficients estimated for each participant were submitted to a weighted least squares (WLS) regression where F-contrasts were computed for the control and parametrically modulated regressors. Scanner type (Trio vs Prisma) was entered as a secondary nuisance regressor. Five thousand permutations of the WLS model were also computed following the Freedman–Lane procedure (Winkler et al., 2014). Every statistical map, both observed and permuted, was submitted to threshold-free cluster enhancement (Smith and Nichols, 2009; Gramfort et al. 2013, 2014) using the recommended parameters (H = 2, E = 0.5, step = 0.1). Finally, the permutation maps were used to compute family-wise error (FWE) corrections (α = 0.05) for each voxel (Winkler et al., 2014). Any resulting clusters were discarded if they covered <100 mm^2^ on the surface or fewer than 20 contiguous voxels of the volume.

### Code accessibility

All data and analysis scripts are available online at https://openneuro.org/datasets/ds001814 and https://github.com/mghneurotherapeutics/DARPA-ARC, respectively. The data and scripts are freely available at these locations with instructions for access and suggested citation included.

## Results

### Behavioral results

Participants exhibited the expected response trends for the ARC task: greater risk of electrical stimulation decreased on average the likelihood of selecting the risky option, whereas increasing reward increased the likelihood of selecting the risky option (Fig. 3). The 95% highest density intervals (HDIs) of the posterior distribution for the group-level parameters showed decreases in risky-choice taking for the 50% (β_1_ = –1.922, 95% HDI: [–2.606, –1.139]) and 90% risk (β_2_ = –4.180, 95% HDI: [–5.273, –3.257]) conditions. In contrast, increases in risky-choice taking were observed in response to increasing reward (β_3_ = 10.652, 95% HDI: [8.239, 12.887]).Thus, risk biased choice behavior toward avoidance (i.e., selecting the safe option), and reward biased choice behavior toward approach (i.e., selecting the risky option), indicating that the ARC task elicited the intended behavioral effects.

At the subject level, the 95% HDIs of the posterior estimates for the 50% risk ($\beta_{1}$) and 90% risk $(\beta_{2}$) coefficients were strictly negative for 19/28 participants and 24/28 participants, respectively. The 95% HDIs for the reward coefficients ($\beta_{3})$ were strictly positive for 27/28 participants. No participants exhibited an increase in choice preference for the risky option with increasing risk and no participants exhibited an increase choice preference for the safe option with increasing reward. In summary, all the participants had response trends that matched our expectations for the ARC task, and most participants’ behavior was modulated by both risk and reward.

**Figure 4.**
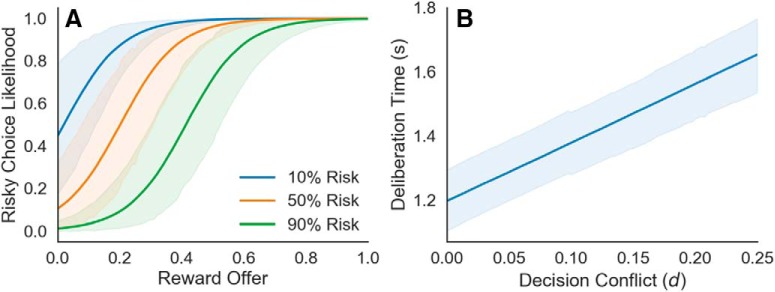
Group-level behavior results. ***A***, The estimated likelihood of choosing the risky option for each risk level and across rewards. The model estimated decreases in risky decision-making at both 50% risk (β_1_ = –1.922, 95% HDI: [–2.606, –1.139]) and 90% risk (β_2_ = –4.180, 95% HDI: [–5.273, –3.257]). In contrast, the model estimated increases in risky decision-making in response to increasing reward (β_3_ = 10.652, 95% HDI: [8.239, 12.887]). ***B***, The estimated linear component of deliberation time as a function of decision conflict, *d*. The model estimated an increase in deliberation time with decision conflict (α_1_ = 0.456, 95% HDI: [0.388, 0.528]). Shaded regions denote the 95% HDI. *Figure Contributions*: Samuel Zorowitz, Katherine Link, and Alexander Rockhill performed the behavioral analysis.

For the response time component of our HBM, we found that approach-avoidance conflict was positively correlated with response times (Fig. 4*B*). At the group-level, the 95% HDI of the posterior distribution on the conflict-RT slope parameter was strictly positive (α_1_ = 0.456, 95% HDI: [0.388, 0.528]). The model estimated an average increase in response times of 0.456 s at maximal conflict. Thus, the ARC task was also successful in eliciting this hallmark behavioral signature of increased response times during approach-avoidance conflict.

**Figure 5.**
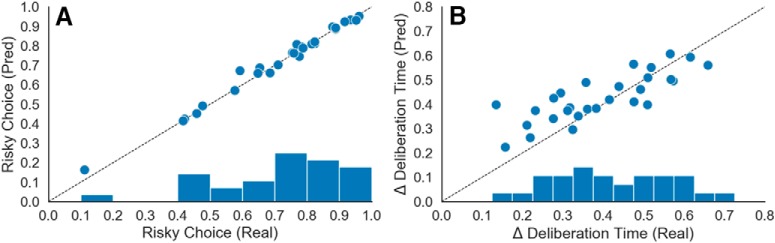
Individual differences in behavior. Participants in the ARC task exhibited large individual differences in behavior. ***A***, Participants varied in their approach-avoidance preferences (although the majority was approach biased). ***B***, Participants varied in the extent to which their deliberation increased in response to decision conflict (but all participants showed increased response times during conflict). Each point represents one participant. The horizontal axis denotes the observed behavior (proportion of risky choices, ***A***; response time increases, ***B***), and the vertical axis denotes the model predicted behavior. Proximity to the diagonal indicates goodness of fit. *Figure Contributions*: Samuel Zorowitz, Katherine Link, and Alexander Rockhill performed the behavioral analysis.

It is important to note we observed considerable variability in the choice preferences of our participants (Fig. 5). The most approach-biased participant selected the risky option on almost all trials (93%), whereas in contrast the most avoidance-biased participant selected the safe option on almost all trials (16%). This strongly demonstrates the notion that the points of maximal approach-avoidance conflict are unlikely to be the same across participants and reinforces the need for methods like HBMs that explicitly take into consideration these large individual differences.

Importantly, posterior predictive checks showed that our model accurately captured participants’ choice behavior (Fig. 5). The root-mean-square error between predicted and observed average risky choice was 0.023. To assess the possibility of model overfitting, we compared the widely applicable information criterion (WAIC; Watanabe, 2013; Vehtari et al. 2017) of our HBM to an equivalent non-hierarchical model (i.e., estimating only group parameters, excluding participant-level parameters). WAIC scores are reported here on deviance scale where lower scores denote greater fitness. The hierarchical model (WAIC = 1319.4) was strongly preferred to its non-hierarchical equivalent (WAIC = 2611.5) despite its greater complexity. We also compared our hierarchical model to a secondary hierarchical model that included interaction terms between risk and reward. This model performed slightly worse than the main effects-only model (WAIC = 1320.8). As such, we proceeded with the more parsimonious model with main effects only for fMRI analysis.

In summary, the ARC task successfully elicited approach, avoidance, and approach-avoidance conflict behaviors from all participants. Specifically, participants were (1) more likely to select the risky option with increasing reward; (2) more likely to select the safe option with increasing risk of electrical stimulation; and (3) slower to respond with increased approach-avoidance conflict. Moreover, participants exhibited large individual differences in their choice preferences, which were accurately captured by our HBM. It is worth reiterating that ignoring these differences can reduce contrast effects in fMRI analysis by averaging over the neural correlates of dissimilar cognitive processes (Ahn et al., 2011).

### Imaging results

**Figure 6.**
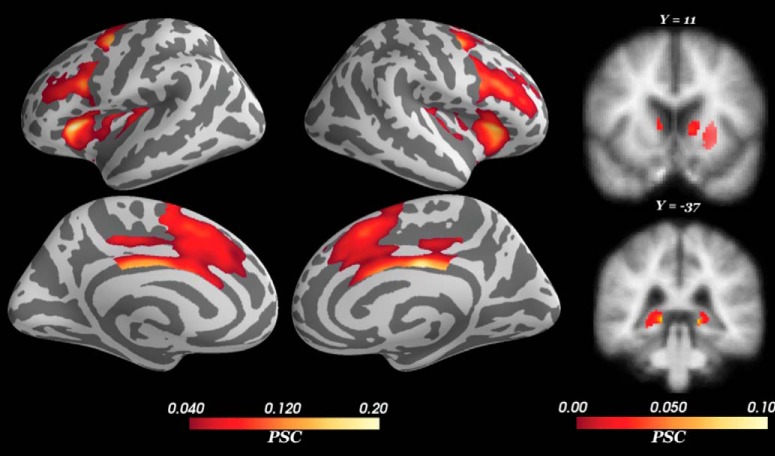
PSC during deliberation. The control regressor measures changes in the BOLD signal during deliberation (independent of approach-avoidance conflict). Positive activation was found in cortical and subcortical regions including the lateral and medial PFC, striatum, and hippocampus. All voxels corrected for multiple comparisons through 5000-iteration permutation testing and voxel-wise FWE corrections (α = 0.05). LH, left hemisphere; RH, right hemisphere. *Figure Contributions*: Samuel Zorowitz and Alexander Rockhill performed the fMRI analysis. Samuel Zorowitz, Alexander Rockhill, and Kristen Kellard collected the data.

For the control regressor (i.e., measuring the average BOLD signal change during the deliberation phase, without modulation by conflict), we found activations within the a priori cortical and subcortical regions of interest (Fig. 6) that were selected based on prior literature (see Results, fMRI modeling and analysis). Peak voxels and their corresponding statistics are reported in Table 1. Large, significant BOLD signal increase was observed in bilateral dorsal anterior cingulate cortex (dACC) and dmPFC (dACC/dmPFC; BA 32), midcingulate cortex (BA 23/24), pre-supplementary motor area (pre-SMA; BA 6), anterior insula (BA 13), and dlPFC (BA 46). Among subcortical structures, the control deliberation regressor was positively correlated with BOLD signal activation in bilateral dorsal hippocampus and striatum (caudate, putamen). Smaller, significant activations were also detected in the right lateral orbitofrontal cortex (OFC; BA 11) and right putamen. These results corroborate the distributed network of neural structures previously reported to be recruited during approach-avoidance conflict tasks (Talmi et al., 2009; Park et al., 2011; Bach et al., 2014; Aupperle et al., 2015; O’Neil et al., 2015; Schlund et al., 2016; Loh et al., 2017).

**Table 1.** Coordinates and statistics of peak BOLD activations

Deliberation phase (control)					
ROI	*x*	*y*	*z*	PSC	*F*
dACC/dmPFC: LH	–12	22	36	0.08	352.92
RH	7	15	24	0.09	462.05
MCC: LH	–7	–22	29	0.15	328.72
RH	7	–15	31	0.18	529.27
pre-SMA: LH	–9	7	51	0.10	419.56
RH	10	14	47	0.10	373.34
dlPFC: LH	–36	9	24	0.12	223.96
RH	36	18	25	0.11	312.87
Anterior insula: LH	–31	27	9	0.2	351.91
RH	31	27	8	0.16	413.00
Lateral OFC: RH	13	38	–24	0.07	95.60
Pre-motor: LH	–37	–2	43	0.14	291.16
RH	36	–3	44	0.14	333.73
Caudate: LH	–10	7	3	0.07	28.42
RH	10	11	5	0.06	25.16
Putamen: LH	–20	5	1	0.05	29.04
RH	34	–7	–7	0.04	22.15
Hippocampus: LH	–14	–39	–3	0.09	34.95
RH	14	–39	–1	0.10	34.47
Deliberation phase (conflict)					
IFG: LH	–39	45	7	0.05	56.53
RH	42	45	–6	0.05	55.10
dlPFC: RH	42	27	31	0.04	68.02
pre-SMA: RH	9	27	46	0.04	59.55

The reported statistics are the PSC and WLSs contrast against baseline (*F*) statistic. The first set of results reflect the unmodulated deliberation and the second set reflect the contrast between deliberation parametrically modulated by conflict and unmodulated deliberation. All coordinates reported in the MNI space and reflect the peak of activation. All voxel statistics were corrected for multiple comparisons through 5000-iteration permutation testing and voxel-wise FWE corrections (α = 0.05). LH, left hemisphere; RH, right hemisphere; MCC, midcingulate cortex.

**Figure 7.**
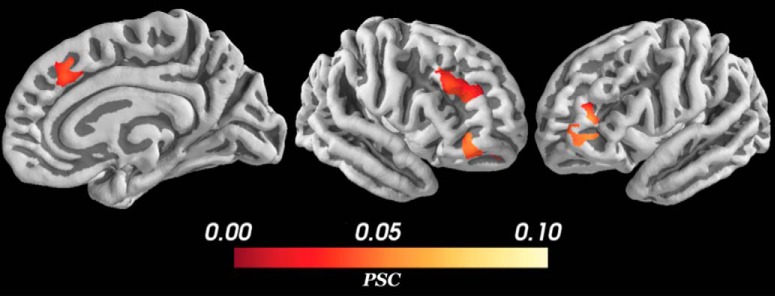
PSC during conflict. The parametric modulation regressor measures changes in BOLD signal during deliberation as a function of approach-avoidance conflict. Positive activation was detected only in bilateral IFG, and right dlPFC, and pre-SMA. All voxels corrected for multiple comparisons through 5000-iteration permutation testing and voxel-wise FWE corrections (α = 0.05). *Figure Contributions*: Samuel Zorowitz and Alexander Rockhill performed the fMRI analysis; Samuel Zorowitz, Alexander Rockhill and Kristen Kellard collected the data.

Significant change in BOLD signal for approach-avoidance conflict regressor was observed in a much more restricted set of structures (Fig. 7). Approach-avoidance conflict was positively correlated with BOLD signal activation only in bilateral rostral inferior frontal gyrus (IFG; pars orbitalis; BA 47), right dlPFC (BA 46), and right dmPFC/pre-SMA (BA 32). No significant positive activations were detected in subcortical structures, and no negative activations were detected in any a priori region of interest. In contrast to the aforementioned previous literature, our results suggest that only a select set of cortical structures tracked approach-avoidance conflict. Interestingly, our analysis revealed conflict representations in the right IFG, a structure previously unreported in the approach-avoidance conflict literature.

**Figure 8.**
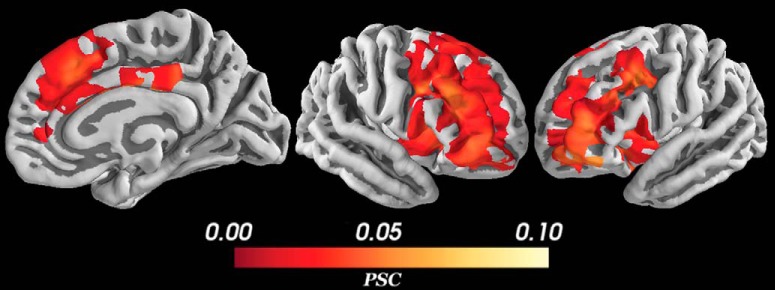
PSC during conflict for the fixed epochs analysis. In this case, epochs were made from the first stimulus presentation to the end of the response period instead of ending when the subject responded for each particular trial. A more widespread, less specific, smaller, positive activation was detected in the same structures as Figure 7 with the addition of activation in bilateral striatum, left insula as well as greater activation in bilateral dlPFC. All voxels corrected for multiple comparisons through 5000-iteration permutation testing and voxel-wise FWE corrections (α = 0.05). *Figure Contributions*: Alexander Rockhill performed the fMRI analysis. Samuel Zorowitz, Alexander Rockhill, and Kristen Kellard collected the data.

The control analyses showed the difference between these results and results from analyses with fixed epochs, averaging across subjects and using a simpler risk or reward only model. As shown in Figure 8, using fixed epochs caused smaller, more widespread, positive activations encompassing bilateral striatum and left insula in addition to the structures activated in the main, variable epochs analysis. The non-HBM (used in combination with variable epochs) had no significant activations that correlated with the conflict regressor. Using risk and reward as regressors in a model with only the non-parametrically modulated, control deliberation regressor and risk or the control deliberation regressor and reward also yielded almost no significant activations with the exception of a small, negative activation correlated with reward in a small area of right dlPFC and lateral OFC. When the risk and reward regressors were modeled in combination with conflict, not only were there no significant activations for the risk and reward regressors, but the significant activations for the conflict regressor was suppressed.

## Discussion

In this study, we investigated the neural basis of human approach-avoidance conflict while accounting for two possible sources of heterogeneity in the literature; individual approach-avoidance variability and time-on-task. Using HBM, we controlled for individual differences in approach-avoidance preference by comparing participants’ fMRI data according to each participant’s relative points of maximal approach-avoidance conflict. Using the variable epochs method in our fMRI analyses, we also controlled for the time-on-task effect. Thus, we were able to differentiate brain structures strictly sensitive to approach-avoidance conflict from those representing information correlated with deliberation more generally. The present findings corroborate previous reports of the anatomic correlates of approach-avoidance behavior by our finding that BOLD signal increased during deliberation across a broad network of cortical and subcortical brain structures (dACC/dmPFC, pre-SMA, dlPFC, OFC, insula, striatum, hippocampus). Importantly, the current findings deviate from the previous literature insofar that our controlled analyses found conflict-related changes in BOLD signal only in a select set of structures (i.e., IFG, dlPFC, and pre-SMA). Collectively, the current findings suggest the importance of careful methodology in isolating the neuroanatomical correlates of latent psychological states such as approach-avoidance conflict.

To examine the effect of using an HBM, we compared these results to results from the non-HBM analysis. The HMB methodology was clearly warranted by the large differences observed between the approach-avoidance behavior of different participants as shown in Figure 5 and described in Results, Behavioral results. The need for this methodology was confirmed by the suppression of any significant areas of activation when a non-HBM was used. Thus, accounting for individual differences with an HBM resulted increased group-level fMRI contrast statistics, consistent with previous findings (Ahn et al., 2011).

Another important difference between the present findings and past studies is our use of the variable epochs method (Grinband et al., 2008), which we included so as to control for the time-on-task effect and minimize the risk of mismodeling the hemodynamic response. By controlling for time-on-task, our analysis was explicitly interested in identifying brain structures that show an increase in the BOLD signal due to an increase in the intensity, not duration, of the activity of the underlying neural populations. One natural question is whether approach-avoidance conflict is more accurately modeled as the prolonged, but not increased, engagement of brain structures. One problem with this view, as noted above, is that this makes it difficult to disentangle conflict-specific signals from other correlated but unrelated signals (e.g., processing of reward or threat stimuli). As such, we opted to use a more conservative definition of approach-avoidance conflict (increase in amplitude of BOLD signal, above and beyond that expected from prolonged engagement, as measured by our parametric modulation regressor). The conservativeness of this variable epochs method compared to fixed epochs was confirmed in the control analyses shown in Figure 8, where areas with significant activation in the variable epochs analysis were found to be a subset of areas with significant activation for the fixed epochs analysis. Thus, our analysis was conservative but well suited to identify regions specifically implicated in the processing of approach-avoidance conflict.

To control for whether our results relate to approach-avoidance and not some simpler approach or avoidance alternative mechanism, we ran three different analyses (1) with risk as the parametrically modulated regressor, (2) with reward as the parametrically modulated regressor, and (3) with three parametrically modulated regressors for risk, reward and conflict. The first two analyses showed that risk or reward alone are not capable of explaining the regions of conflict that had significant activations correlated with conflict (Fig. 7); as described in Results, these analyses had almost no areas of significant activation. In the third analysis, the suppression of significant conflict activations (described in Results) suggested that including risk and reward in the same model as conflict caused the variance to be split between all three variables’ explanatory power. Reward and risk are approach and avoidance stimuli, respectively, so by definition these stimuli covary strongly with the approach-avoidance measure conflict. This control analysis therefore confirms that the explanatory power of conflict is dependent on risk and reward and also shows that including regressors with high covariance can cause a false-negative result.

Another point worth noting is that our analysis assumes only linear changes in the BOLD response to conflict. The variable epochs method used here is insensitive to any nonlinear changes in the BOLD signal that may arise as a function of response time, raising the possibility of remaining biases in the present results. Interestingly, in a finite impulse response analysis of the hemodynamic response during prolonged response times, Yarkoni et al. (2009) found that structures in the PFC were better described by increases in the amplitude of hemodynamic response but not by changes in its shape. These findings suggest that not using a finite impulse response analysis did not bias the hemodynamic response in this present analysis, but further studies are necessary to answer this question more definitively.

There were additional discrepancies between the present study and previous studies on approach-avoidance tasks. Though positive BOLD activation was detected during deliberation in the right OFC, the effect was considerably smaller than previously reported findings (Talmi et al., 2009; Schlund et al., 2016). This may reflect signal-to-noise ratio issues particular to surface-based analysis of the OFC (Stenger, 2006). Additionally, in contrast to Schlund et al. (2011) and Aupperle et al. (2015), amygdala activation was not detected during deliberation. In this study, suboptimal calibration of the stimulation amperage likely diminished participants’ perception of threat from the stimulation and consequently their amygdala activation. Finally, the bilateral hippocampus activations detected during deliberation were located dorsally, rather than anteriorly/ventrally as have been previously reported in literature on threat processing (Bach et al., 2014). The dorsal hippocampus has been associated with cognition and planning (Fanselow and Dong, 2010), so these activations could reflect participants’ processing of the conditional structure of the ARC task (e.g., “if *safe* is chosen, then 0% chance of electrical stimulation; if *risky* is chosen, then X% chance of electrical stimulation”).

This study had several limitations. Due to the equipment issues described above, as well as the use of non-adaptive rewards, we were unable to calibrate the reward and risk of the ARC task according to each participant’s choice preferences. This may be one reason why we observed an approach-bias on average. This also means that the present study undersampled trials at or near the points of participants’ maximal approach-avoidance conflict. A consequence of this undersampling is that many of the high conflict decisions participants made in this task occurred during high risk trials, making it harder to divorce conflict from risk. Future approach-avoidance conflict experiments should consider incorporating adaptive design optimization (Myung et al., 2013) to titrate the levels of rewarding and threatening stimuli according to future participants’ choices preferences to minimize the influence of these potential biases.

Finally, it is worth noting that the set of structures we found correlated with approach-avoidance conflict (i.e., IFG, dlPFC, and pre-SMA) share overlap with the putative response inhibition network (Aron et al., 2004, 2014; Aron and Poldrack, 2006). One interpretation of the present results is that approach-avoidance conflict is another process requiring response inhibition, wherein the IFG inhibits prepotent motor responses to facilitate prolonged evidence accumulation during difficult choices. This interpretation is consistent with the increased response times observed in the present experiment. The possible role of the inhibition network during approach-avoidance conflict points to a clear direction for future studies; investigating whether the putative response inhibition network works to signal response conflict to other brain structures, such as through the hyperdirect pathway to the basal ganglia (Frank et al., 2015). Alternately, these structures may be involved in the resolution of approach-avoidance conflict, such as by biasing choice toward approach or avoidance. In either case, the framework that this study presents for the consideration of individual-level behavioral variation and the time-on-task effect would likely lead to benefits in specificity and accuracy of future studies investigating similar cognitive processes.

## Synthesis

Reviewing Editor: Philippe Tobler, University of Zurich Decisions are customarily a result of the Reviewing Editor and the peer reviewers coming together and discussing their recommendations until a consensus is reached. When revisions are invited, a fact-based synthesis statement explaining their decision and outlining what is needed to prepare a revision will be listed below.

Dear Prof. Deckersbach,

Thank you for transmitting your revised manuscript to eNeuro. While the revision has addressed some of the points raised by the reviewers, two points remained insufficiently addressed.

In relation to the second point of reviewer 1, the rebuttal states “If particular areas lose their significance because of the accounting for the confound of risk-reward valuation and others by accounting for response time, those would be interesting results but ultimately would not inform the findings that remained significant, which is the focus of our results.” In contrast to this assertion, the paper states: “We found that using these methods increased the specificity of the structures responding to conflict.” This conclusion would be backed up by analysis much more directly if the suggestion of the reviewer would be implemented. Indeed, the authors implicitly predict that not accounting for individual differences should result in fewer conflict-related regions whereas not controlling for response times should result in more conflict-related regions. It would make the paper stronger to directly test these two hypotheses.

In relation to the fourth point of reviewer 2, the paper uses reward and risk to create approach-avoidance conflicts. In order to make conclusions about such conflicts over and above the specific task used, it is essential to control for both reward and risk, showing that the results are not due to either of these through more stringent control analyses. Otherwise the readers will be left wondering whether the paper really is about conflict or a fairly obvious alternative.

## Author Response

--------------------------------------------

Manuscript Instructions

---------------------------------------------

Synthesis of Reviews:

Computational Neuroscience Model Code Accessibility Comments for Author (Required):

N/A

Significance Statement Comments for Author (Required):

N/A

Comments on the Visual Abstract for Author (Required):

N/A

Synthesis Statement for Author (Required):

Thank you for transmitting your revised manuscript to eNeuro. While the revision has addressed some of the points raised by the reviewers, two points remained insufficiently addressed.

In relation to the second point of reviewer 1, the rebuttal states “If particular areas lose their significance because of the accounting for the confound of risk-reward valuation and others by accounting for response time, those would be interesting results but ultimately would not inform the findings that remained significant, which is the focus of our results.” In contrast to this assertion, the paper states: “We found that using these methods increased the specificity of the structures responding to conflict.” This conclusion would be backed up by analysis much more directly if the suggestion of the reviewer would be implemented. Indeed, the authors implicitly predict that not accounting for individual differences should result in fewer conflict-related regions whereas not controlling for response times should result in more conflict-related regions. It would make the paper stronger to directly test these two hypotheses.

In relation to the fourth point of reviewer 2, the paper uses reward and risk to create approach-avoidance conflicts. In order to make conclusions about such conflicts over and above the specific task used, it is essential to control for both reward and risk, showing that the results are not due to either of these through more stringent control analyses. Otherwise the readers will be left wondering whether the paper really is about conflict or a fairly obvious alternative.

-

Response:

To directly test the first two hypotheses, as suggested by the reviewer, we ran additional analyses with the same process. In the first supplemental analysis, the epochs were fixed to the total time available for the subject to respond while the hierarchical Bayesian model was used. In the second, the non-hierarchical Bayesian model was used but epochs were of variable length.

For the first comparison, of fixed epochs compared to variable epochs, the control regressor had more significant regions of correlation that survived cluster permutations for variable epochs than for fixed epochs. We think this is likely because the deliberation was occurring exclusively before the selection of choice. Fixed epochs include times (after convolution with the hemodynamic response function) after this period. We believe this introduces noise from separate processes, for example related to anticipation of receiving a shock.

Figure 1. Variable epochs control regressor. Positive activation was found in cortical and subcortical regions including the lateral and medial prefrontal cortex, striatum, and hippocampus as described in Table 1 of the paper.

Figure 4. Fixed epochs control regressor. Positive activations were found in a subset of the areas found in Figure 1; the lateral prefrontal cortex, striatum, and hippocampus.

In line with this explanation that signal from cognitive processes outside the deliberative period may have been included when using the fixed epochs regression analysis, there were similar but more widespread correlations for the fixed epochs analysis distance-to-the-decision-boundary (DDB) regressor. This is consistent with the reviewer's point that out paper implies that “not controlling for response times should result in more conflict-related regions,” but now shows direct evidence for this claim as suggested by the reviewer.

Figure 5. Variable epochs distance-to-the-decision boundary regressor. Positive activation was detected only in bilateral inferior frontal gyrus (IFG), and right dorsolateral prefrontal cortex (dlPFC) and pre-supplementary motor area (pre-SMA).

Figure 6. Fixed epochs distance-to-the-decision boundary regressor. Positive activation was detected in a superset of regions compared to the variable epochs analysis with the addition of activation in bilateral striatum, left insula as well as greater activation in bilateral dlPFC.

In order to test the prediction that “not accounting for individual differences should result in fewer conflict-related regions,” we used an analysis with a non-hierarchical Bayesian model. Here, individual conflict estimates were drawn from a maximally-likely group distribution agnostic to individual-level variability. The DDB regressor for both fixed and variable epochs had no significant correlations that survived multiple comparison cluster corrections. This provides direct evidence that the hierarchical model offers greater statistical power.

Figure 7. Non-hierarchical variable epochs distance-to-the-decision boundary regressor. No significant clusters after cluster permutation correction.

Figure 8. Non-hierarchical fixed epochs distance-to-the-decision boundary regressor. No significant clusters after cluster permutation correction.

In order to test the last hypothesis suggested by the reviewer that our results are really “about conflict [instead of] a fairly obvious alternative”, we ran analysis with risk as the only regressor (other than the non-parametrically regressor), reward as the only regressor, and risk and reward as co-regressors to distance-to-the-decision boundary. Risk and reward by definition covary strongly with distance-to-the-decision boundary and thus controlling for these factors without causing a false-negative required multiple approaches.

The analysis with risk or reward as the only regressors showed almost no areas to have significant correlations after cluster permutation correction. This is direct evidence that risk or reward alone are not capable of explaining the regions of conflict found in this paper with the DDB model.

Figure 9. Risk regressor. No significant clusters after cluster permutation correction.

Figure 10. Reward regressor. Small areas of negative correlation were found in the right dlPFC and lateral orbitofrontal cortex.

To test whether controlling for risk and reward yielded the areas of conflict found in the original analysis, we included regressors for risk and reward in the same generalized linear model (GLM) as DDB. This dramatically suppressed significant clusters; no regions had any significant correlations with risk, reward or DDB after cluster permutation correction. One likely explanation for this may be the covariance of risk and reward with DDB. Although the risk only and reward only models did not have any significant correlations with the BOLD signal, they still are partially colinear with DDB. Thus, when all three are included in a regression, the variance is split between all three variables' explanatory power. This “soaking up” of variance left no significant areas that were correlated with DDB after cluster permutation correction. While some covariance between risk, reward and DDB is inevitable because an approach-avoidance measure must necessarily covary with both approach and avoidance, the sampling of risks and rewards could be improved to more evenly cover approach-avoidance space. This shortcoming is explained in the paper, as are the merits of this potential improvement.

Figure 11. Distance-to-the-decision boundary regressor with risk and reward co-regressors. No significant clusters after cluster permutation correction.

Figure 12. Reward regressor with risk and DDB co-regressors. No significant clusters after cluster permutation correction.

Figure 13. Risk regressor with reward and DDB co-regressors. No significant clusters after cluster permutation correction.

---------------------------------------------

Manuscript Instructions

---------------------------------------------

Synthesis of Reviews:

Computational Neuroscience Model Code Accessibility Comments for Author (Required):

N/A

Significance Statement Comments for Author (Required):

N/A

Comments on the Visual Abstract for Author (Required):

N/A

Synthesis Statement for Author (Required):

Reading the paper, I noticed a few issues:

Line 227: an in the increase -> an increase

Response: The change was duly corrected, thank you for pointing this typo out.

Line 230: “The amplitude of the parametric modulation term was linearly scaled according to the level of risk (i.e. 10%, 50%, 90%)” is unclear, given that above (line 220) the parametric modulator was described as scaling with conflict. Is risk a second, separate parametric modulator? Please clarify (and possibly move some of the methods description regarding the control analyses from the Discussion (lines 377-380) to the Methods)

Response: That parametric modulator was used in one of the control analyses and not in the main analysis, which was unclear, thank you for pointing out that mistake in organization. That part of the Methods section has been clarified, and the descriptions of control analyses were moved from the Discussion to the Methods section as is more appropriate as suggested.

Line 258: decreases risky-choice taking -> decreases in risky-choice taking

Response: Thank you for pointing out this error, it has been duly corrected.

Lines 208-209, 307-308: It is unclear which prior literature served to define regions of interest. Please specify

Response: The prior literature was described in lines 42-44 in the introduction, but the reviewer makes a very good point that this was not clear that these references were supposed to apply to the later instances. This has been clarified by referencing the studies whose findings led to our choice of a priori regions of interest, thank you for improving the clarity of the paper by pointing this out.

Line 335 (and 8 further instances): it is unclear what “DDB” stands for. Preferably avoid this abbreviation throughout

Response: Apologies for changing acronyms, DDB stands for distance-to-decision-boundary as described on lines 159-161. It is synonymous and the definition of our conflict measure so “DDB” was changed to conflict. Thank you for pointing that out and sorry for the confusion.

Lines 354-355: Somehow the distinction between the specific and less specific form of conflict in that sentence should become clearer (it may help to add “more generally” after “from those representing information correlated with conflict” but it would be better to be even more explicit in what you mean with “information correlated with conflict”)

Response: To address this point, “information correlated with conflict” was changed to “information correlated with deliberation more generally” so as to clarify the difference between the control regressor and the parametrically modulated regressor. Here, deliberation during a task involving conflict is implicit definition of less specific conflict whereas the signal associated with trials involving greater conflict is the more specific conflict. Thank you for catching this inconsistency, the meaning is now much less ambiguous.

Line 425: delete “share”?

Response: Thank you for correcting that, more appropriate wording has been adopted.
